# What does it mean to be affiliated with care?: Delphi consensus on the definition of “unaffiliation” and “specialist” in sickle cell disease

**DOI:** 10.1371/journal.pone.0272204

**Published:** 2022-11-11

**Authors:** Andrea E. Lamont, Lewis L. Hsu, Sara Jacobs, Robert Gibson, Marsha Treadwell, Yumei Chen, Richard Lottenberg, Kathleen Axelrod, Taniya Varughese, Cathy Melvin, Sharon Smith, Ifeanyi Beverly Chukwudozie, Julie Kanter, the Sickle Cell Disease Implementation Consortium

**Affiliations:** 1 Wandersman Center, Columbia, SC, United States of America; 2 University of Illinois, Chicago, Illinois, United States of America; 3 RTI International, Research Triangle Park, NC, United States of America; 4 Medical College of Georgia, Augusta University, Augusta, GA, United States of America; 5 UCSF School of Medicine, UCSF Benioff Children’s Hospital Oakland, Oakland, CA, United States of America; 6 UCSF Benioff Children’s Hospital Oakland, Oakland, CA, United States of America; 7 University of Florida, Gainesville, FL, United States of America; 8 Washington University St Louis, St. Louis MO, United States of America; 9 Medical University of South Carolina, Charleston, SC, United States of America; 10 National Heart Lung and Blood Institute (NHLBI), National Institutes of Health (NIH), Bethesda, MD, United States of America; 11 University of Alabama, Birmingham, Birmingham, AL, United States of America; PLOS ONE, UNITED KINGDOM

## Abstract

Accruing evidence reveals best practices for *how* to help individuals living with Sickle Cell Disease (SCD); yet, the implementation of these evidence-based practices in healthcare settings is lacking. The Sickle Cell Disease Implementation Consortium (SCDIC) is a national consortium that uses implementation science to identify and address barriers to care in SCD. The SCDIC seeks to understand how and why patients become unaffiliated from care and determine strategies to identify and connect patients to care. A challenge, however, is the lack of agreed-upon definition for what it means to be unaffiliated and what it means to be a “SCD expert provider”. In this study, we conducted a Delphi process to obtain expert consensus on what it means to be an “unaffiliated patient” with SCD and to define an “SCD specialist,” as no standard definition is available. Twenty-eight SCD experts participated in three rounds of questions. Consensus was defined as 80% or more of respondents agreeing. Experts reached consensus that an individual with SCD who is unaffiliated from care is “someone who has not been seen by a sickle cell specialist in at least a year.” A sickle cell specialist was defined as someone with knowledge and experience in SCD. Having “knowledge” means: being knowledgeable of the 2014 NIH Guidelines, “Evidence-Based Management of SCD”, trained in hydroxyurea management and transfusions, trained on screening for organ damage in SCD, trained in pain management and on SCD emergencies, and is aware of psychosocial and cognitive issues in SCD. Experiences that are expected of a SCD specialist include experience working with SCD patients, mentored by a SCD specialist, regular attendance at SCD conferences, and obtains continuing medical education on SCD every 2 years.” The results have strong implications for future research, practice, and policy related to SCD by helping to lay a foundation for an new area of research (e.g., to identify subpopulations of unaffiliation and targeted interventions) and policies that support reaffiliation and increase accessibility to quality care.

## Introduction

Sickle cell disease (SCD) is an inherited group of blood disorders characterized by an abnormal form of hemoglobin molecule in red blood cells that transport oxygen throughout the body [1]. It is a severe and debilitating illness that can substantially affect sufferers’ length and quality of life. Currently, SCD affects an estimated 100,000 individuals living in the United States, with a shortage of adult-oriented providers trained in SCD to sufficiently meet their needs [2,3]. As a result, many adults with SCD are not regularly seen by a provider with knowledge of best clinical practices in SCD; instead, they receive exclusively episodic acute care, often in the emergency department (ED) [4]. In the absence of any national public health surveillance tracking system or registry of individuals with SCD, the extent of the problem of lack of comprehensive care by SCD expert physicians is currently unknown. Since the therapeutic scenario is evolving with new treatments regularly available, there is a need for increased empirical attention on individuals with SCD not affiliated with care.

There is very little information available about the subpopulation of individuals with SCD without comprehensive care from a research perspective. While some information is known about certain subpopulations of individuals with SCD not receiving care (e.g., those who appear in the ED or those that were lost during transition to adult care), the scope of the problem of patients unaffiliated with care has not been systematically examined. Part of this lack of knowledge is that the term “unaffiliated” has not been well defined for SCD as it has in other rare diseases. We anticipate that this is a heterogeneous group of individuals, but lack of consensus about definitional terms has prevented forward movement in developing diverse interventions to find and connect different subpopulations of unaffiliated individuals with care. The purpose of this study was to better define what it means to be unaffiliated from SCD care and what it means to be an SCD specialist. Using these definitions, we can better design and quantify the impact of interventions to improve this public health deficit in the future.

### The problem Of unaffiliation

Although a relatively rare disease, SCD places a heavy burden on the health care system [5,6]. Approximately $488 million was spent on SCD-related expenditures in the year 2004 alone, much higher than the general population [5,6]. One driver underlying high costs is the frequency of hospitalizations for SCD-related illnesses [7,8]. Patients with SCD have an estimated 7–30 times higher rates of hospitalization and 2–6 times higher rates of ED usage than the general population [9]. Overreliance on EDs or hospitalization is a concern for both quality and cost-efficient care of SCD. Proper management of SCD requires evidence-based comprehensive and preventive care in an outpatient setting [10,11] coupled with availability of an infusion center or ED for management of the acute complications associated with SCD. Because sequential outpatient preventive care is not designed to be provided within the ED system, heavy reliance on ED services over a comprehensive care approach may obstruct optimal clinical outcomes [9,12].

In 2014, guidelines for the care of SCD were released by the National Heart, Lung, and Blood Institute’s (NHLBI’s) 2014 Expert Consensus Panel. Guidelines were also recently published by the American Society of Hematology (ASH) [10], although the timing of publication prevented their inclusion in this study. Together, these two sets of guidelines are used by experts to inform the standard of care [10,11]. Guidelines help improve quality of care by improving clinical decision-making and expediting new scientific and therapeutic advances. The NHLBI and ASH SCD guidelines emphasize that SCD comprehensive care and preventive care are delivered in the non-acute, ambulatory setting.

The guidelines were intended to improve care by supporting physicians in treating patients with SCD. Yet, despite efforts to disseminate guidelines, many physicians are unaware of their existence [13]; and, when they are aware, they often do not show adherence [14]. There are patient-, provider-, and system-level barriers to explain this implementation gap (i.e., the gap between what is known to be effective in research and what is implemented in practice). For example, barriers exist when implementing guidelines in a standard, nonspecialist primary care setting because insurance companies commonly do not reimburse for certain types of difficult and time-consuming activities outlined in the guidelines. Moreover, many providers do not have access to SCD specialists for support. This results in individuals with SCD receiving sub-optimal care, despite being seen by a physician. Even when physicians knowledgeable of the guidelines do exist in a given geographical area, a significant proportion of individuals with SCD do not have access to these specialists (e.g., due to transportation, insurance concerns, and other barriers) or have inconsistent clinic attendance (and therefore do not receive the benefits of the care). Inconsistent attendance at specialty clinics is likely a multifactorial problem and an active area of empirical inquiry [15]. Regardless of the cause, unaffiliation from SCD-specific care often results in preventable acute care encounters, including the ED or hospitalization [16]. It is very hard to implement evidence-based interventions in these contexts, and therefore patients end up with unmet needs and poorer clinical outcomes.

From a research perspective, patient overreliance on the emergent or acute care system (versus a comprehensive care approach) poses significant concerns for how we understand and treat SCD. Since the population of patients who are unaffiliated from the SCD preventative care system are not well defined, we do not know the extent to which those individuals who do not receive care or only receive acute care resemble those who regularly receive comprehensive care by an expert physician. Existing SCD studies tend to draw from the populations of individuals with SCD that the researchers could access, which are typically not those unaffiliated from the system. This means that we do not know the extent that existing knowledge on SCD applies to those who are unaffiliated from care. Existing studies may be biased by the availability and accessibility of SCD patients willing to participate in the research. While the population of interest is “all individuals with SCD”, samples used are “patients with SCD involved in comprehensive care”.

There is no current definition of “unaffiliation” in the SCD field nor a uniform definition as there is in other complex chronic disease fields like HIV, mental health, diabetes, or juvenile rheumatoid arthritis. In other fields, patients who “disengaged,” “dropped out,” “abandoned care,” or were “out of care” or “lost to follow-up” are defined with both the type of care missed and a time frame without appointments, ranging from 6 months [17,18] to 1 year [19,20] to 2 years [21–23]. The lack of uniform definitions in SCD creates a problem for researchers: interventionists cannot target a group of people without a clear definition of whom they were targeting.

### Sickle cell disease implementation consortium

In 2016, the National Heart, Lung, and Blood Institute funded the Sickle Cell Disease Implementation Consortium (SCDIC) to address implementation barriers to the provision of high-quality care for SCD [24]. This funding represents a major opportunity for system-level change in relation to SCD. It is the first research program to use implementation science (defined as “the scientific study of methods to promote the systematic uptake of research findings and other evidence-based practices into routine practice, and, hence, to improve the quality and effectiveness of health services” [25]) to identify and address barriers to care for SCD. SCDIC is an eight-site consortium of academic medical centers with specialized sickle cell treatment centers across the United States, comprising both sickle cell experts and implementation scientists collaborating to improve the quality of sickle cell care. An initial goal of SCDIC was to ensure that individuals with SCD receive guideline-based SCD care. However, it became difficult to assess what constituted an SCD-specialist, which slowed the movement in the consortium’s ability to identify and connect unaffiliated patients to care. Thus, a primary aim (among others described elsewhere [24]) of the consortium is to understand why affected individuals are not affiliated with SCD clinics or expert providers and to reconnect them with high-quality care.

At the start of the project, the SCDIC Unaffiliated Patients Working Group had proposed a working definition: “a person with SCD who has not seen an adult SCD specialist for ambulatory clinic in 2 years.” This definition was regarded as searchable in administrative databases, and 2 years without an outpatient visit would mean that guideline-based annual screenings would certainly have been missed. However, the working group soon realized through conversations with experts and patient stakeholders that there was substantial disagreement with different aspects of this definition, specifically regarding the time since the last outpatient visit and the type of provider required. Variations in perspectives were the result of different experiences, roles, and familiarity with different patient populations (e.g., rural settings where access to a physician with expertise in SCD is unavailable versus urban settings where unaffiliation may be for other reasons). There was also disagreement over what constitutes an “SCD specialist” provider. For example, key stakeholders and experts discussed whether a primary care physician could be a specialist (or only hematologists), as well as the types of experiences, training, and knowledge required to be a specialist. Unifying the different perspectives across experts with diverse experiences with SCD presented a challenge with the nuances of the definition. This was the stimulus for the current consensus building process.

### The present study

Because the study of “unaffiliation” in the context of SCD is relatively new, a clear definition was needed to shape the field in a meaningful way. This study was conducted to use a systematic consensus-building process to obtain the definition of unaffiliation. We wanted to ensure that all expert perspectives were taken into consideration so that the foundational definitions were not biased by a few individuals on the project. We aimed to capitalize on the breadth of knowledge and experiences from both researchers on the project and clinical practitioners, guideline (best practices) developers, and funders, along with experts with representation from professionals in diverse settings and patient populations. The primary objective of this study was to conduct a Delphi consensus-building process to decide on a working definition of “an unaffiliated patient with SCD.” We specifically sought agreement in the following overarching domains: (a) the type of providers patients need to see to be considered affiliated; (b) the requirements to be considered a SCD specialist; and (c) the length of time out of care to be considered unaffiliated.

## Methods

As part of the SCDIC, we conducted a Delphi process with people with professional expertise in SCD (i.e., researchers, practitioners, funders) to obtain consensus on terms related to “unaffiliation” from SCD. Details of our purposeful sampling appear below. The Delphi technique, primarily developed by Dalkey and Helmer [26] at the Rand Corporation in the 1950s, is a widely used and validated method of obtaining consensus about real-world knowledge solicited from experts within certain topic areas [27]. It involves multiple rounds of questioning via questionnaires to obtain collective opinions of experts until a consensus is reached. In this study, we used 80% agreement as evidence of consensus. Three rounds of surveys were planned and sent out electronically. Reminders were sent to increase participation about once a week (a total of approximately three reminders each round). We did not collect names or affiliations with surveys; however, information about the work setting and other identifiable factors (see below) were collected, rendering the survey confidential but not completely anonymous.

The Delphi survey was developed collaboratively by the SCDIC workgroup of SCD experts and implementation scientists. We used the following working definition as a stimulus for the development of the survey items: “An unaffiliated patient is a person with SCD who has not seen an adult SCD specialist for ambulatory clinic in 2 years.” We obtained input from a group of eight adults living with SCD (stakeholders in SCDIC) on the survey and response items; in particular, this group provided input on the duration of time since last visit, with 12 months (instead of the 2 years in the original definition) selected as the benchmark to correspond to annual check-ups. This group was used to ensure that the patient perspectives were included in the study design. The format of these discussions was open-ended and collaborative in nature. The final survey was approved by the broader SCDIC leadership, including NHLBI representatives not participating in the study.

In round 1, a set of 16 items (including 4 demographic questions) were included that asked participants to rate agreement on a set of items related to definitional terms related to “unaffiliation” and SCD experts. Most items were scored as a “yes/no” or multiple choice format. Open-ended questions were also included to elicit ideas related to unaffiliation that may have been missed. In rounds 2 and 3, items that did not reach consensus in the previous round were resent to participants. Round 2 consisted of 14 questions (including 4 demographic questions); round 3 consisted of 11 questions (including 4 demographic questions). Results of the previous round were also provided for each item. This is standard in a Delphi technique to help move participants toward consensus. Additional items were added to each round that reflected contributions from the open-ended questions.

At the end of the Delphi, we conducted a teleconference debriefing focus group where all participants were invited to participate. During this call, we shared results and facilitated targeted discussions of the implications of the findings. This helped us obtain clarity over some items and deepen our understanding of the quantitative results.

This study was approved through the Institutional Review Board of the University of Illinois, Chicago. Surveys were sent out via email directly from the research team to the invited participant. Informed consent was provided; consenting participants clicked a link that directed them to a confidential survey to begin.

### Participants

Participants of this study were specifically selected based on their range of knowledge and experience working with SCD. We used a purposeful sample of researchers, practitioners, and NHLBI scientists. We intended to sample from the following non–mutually exclusive areas: at least two of the original authors of the NHLBI 2014 guidelines, two representatives from NHLBI, three members of the workgroup within SCDIC focused on unaffiliation (these participants were not part of the planning or analysis of this study), at least 10 clinicians who work with SCD patients, and at least eight investigators from the SCDIC consortium, plus additional investigators recognized in the SCD field who fit a need from the other criteria for our purposeful sample (e.g., working with rural populations, primary-care specialists who specialize in SCD). Our final sample appears in the next section. Additionally, we sought representation from the following domains: role (researcher, policy level, practitioner, developer of guidelines), primary setting (urban, rural), population expertise (adult/pediatric), work setting (SCD center, ED, primary care, non–SCD center hematology), involvement with SCDIC (involved as an investigator or core team member, not involved). Recruitment of participants occurred via direct email to potential participants.

## Results

Twenty-eight experts were invited to participate in the Delphi process through this selection strategy. All participants were MDs, except for one nurse/researcher and one nurse practitioner. Characteristics of respondents appear in Table 1.

**Table 1:** Characteristics of participants.

	Round 1 (n = 21)	Round 2^1^ (n = 16)	Round 3 (n = 20)
**Primary Role** ^**2**^			
Researcher (general)	5%	6%	6%
Researcher (focus sickle cell disease)	36%	34%	40%
NIH leadership	0%	3%	0%
Guidelines developer	19%	19%	18%
Provider	40%	38%	36%
**Work Setting**			
Urban	75%	69%	84%
Rural	5%	6%	0%
Suburban	10%	19%	11%
Other	10%	6%	6%
**Target Population (Patients)**			
Adults	50%	44%	56%
Children	25%	19%	17%
Both	25%	38%	28%
**Proportion of Time Working with SCD**			
Less than 50%	45%	44%	32%
Greater than 50%	55%	56%	68%

^1^ In round 2, there were 19 responses with 3 with complete nonresponse. In Round 3, there were 20 responses with 1 complete nonresponse.

^2^ Respondents were asked to indicate their primary role if they had dual roles. Only one response option could be selected.

### What is an unaffiliated patient?

Results from the three rounds of the Delphi process appear in Table 2. Our first line of inquiry focused on the type of provider a patient must see to be considered affiliated. By round 2, consensus was reached that for a patient to be affiliated, a person with SCD must be seen by an SCD specialist (62% round 1; 88% in round 2). We define what it means to be an SCD specialist in the next section.

**Table 2:** Results of the Delphi survey items related to defining unaffiliation.

*Question*: *For a person with sickle cell disease to be affiliated*, *do they need to see a sickle cell specialist?*			
	**Round 1**	**Round 2**	**Round 3**
Yes	62%	88%	N/A
No	35%	13%	N/A
Unsure	5%	0%	N/A
*Question*: *What type of provider must a person with sickle cell disease see to be affiliated?*			
	**Round 1**	**Round 2**	**Round 3**
Sickle Cell Disease Specialist	65%	88%	N/A
Primary Care Physician	10%	0%	N/A
Advanced Provider (AP) in a Hematology Office	5%	0%	N/A
AP in an PCP office	20%	13%	
*Question*: *A person with sickle cell disease should be considered unaffiliated if the person has not been seen by a provider outside the emergency department or urgent care setting in*:			
	**Round 1**	**Round 2**	**Round 3**
More than 6 months	0%	N/A	N/A
More than a year	76%	75%	89%
More than 2 years	10%	25%	11%
More than 3 years	5%	0%	0%
Unimportant	5%	0%	0%
Other	5%	N/A	N/A
*Question*: *When do you consider a patient lost to follow-up (e*.*g*., *no care follow-up*, *missed appointments)?*			
	**Round 1**	**Round 2**	**Round 3**
More than 6 months	5%	N/A	N/A
More than a year	52%	63%	53%
More than 2 years	38%	31%	47%
More than 3 years	5%	6%	0%
Unimportant	0%	0%	0%
Other	0%	N/A	N/A

The second line of inquiry related to the definition of unaffiliation was the duration of time out of care before a patient is considered unaffiliated. In round 3, experts reached consensus that a person with SCD is considered unaffiliated if they were not seen by any SCD specialist in more than a year. In further conversations with the SCDIC and experts in the field, however, nuances related to this issue continued to be deliberated. Specifically, experts discussed the issue of unaffiliation due to being lost to follow-up and the amount of time needed between appointments before an individual is considered unaffiliated. While it was clear that a lack of contact with a provider (out of care) in a year led to the determination of “unaffiliation”, there was less agreement regarding time between appointments and the designation of unaffiliation. Some experts felt that a year between appointments was sufficient to be considered “lost to follow-up” (and therefore unaffiliated); others felt that the time between appointments should be two years before determining “lost to follow-up” (and therefore unaffiliated). Consensus on the amount of time between appointments before a person is considered unaffiliated as a result of being lost to follow-up was not reached.

Last, we probed whether there are characteristics of patients that should be considered in defining unaffiliation, beyond the type of provider seen and duration of time since last appointment. In rounds 1 and 2, we asked if there were other factors beyond those on the survey that should be considered when defining an unaffiliated SCD patient. Responses to these questions were included in the round 3 survey (see Table 3). The only item that reached consensus was not getting recommended routine SCD screenings. Further, there was consensus in round 1 that there are no exclusions when considering unaffiliation (81%), including healthy individuals who rarely or never get pain or need hospitalization (0% responded that this population can be excluded), adolescents who aged out of pediatrics and have been lost to follow-up (5% responded that this population can be excluded), patients dismissed from care for non-adherence (5% responded that this population can be excluded), and patients dismissed for another reason (10% responded that this population can be excluded).

**Table 3:** Additional factors that should be considered in the definition of an unaffiliated patient.

*Question*: *Should each of the following factors be considered in the definition of an unaffiliated person with sickle cell disease?* *(yes/no; % yes reported)*	
Routine use of emergency department services	63%
Not getting recommended routine sickle cell disease screenings	89%
Proportion of use of inpatient to outpatient care	47%
Location and distance from a sickle cell disease specialist	47%
A joint model of multiple providers (e.g., sickle cell disease specialist and hematology/oncology trained provider in the interim)	58%
Patient knowledge of disease and necessary care	26%

### What is an SCD specialist?

To help understand the type of care a patient must receive to be considered affiliated, we asked participants, *“What types of knowledge and experience must a provider have in order to be considered a SCD expert?”* Results appear in Table 4. To be considered a sickle cell specialist, consensus was reached on the following criteria: knowledge of the 2014 NIH Guidelines, “Evidence-Based Management of SCD”; experience working with SCD patients; has been mentored by an SCD specialist; regularly attends SCD conferences, trained in pain management; trained on SCD emergencies; trained on hydroxyurea management; trained on transfusions; trained on screening for organ damage in SCD; and awareness of psychosocial and cognitive issues in SCD. There was agreement that continuing medical education in SCD specific issues should be obtained at least every 2 years.

**Table 4:** Characteristics of an SCD specialist.

*Question*: *What type of knowledge/training and experience must a provider have to be considered a sickle cell specialist? (yes/no)*^1^				
	**Round 1**		**Round 2**	**Round 3**
Knowledge of the 2014 NIH Guidelines, "Evidence-Based Management of SCD"	90%		N/A	N/A
Experience working with sickle cell disease	95%		N/A	N/A
Experience on sickle cell disease research studies	24%		31%	26%
Mentored by a sickle cell disease specialist	52%		93%	N/A
General Fellowship experience sufficient	20%		27%	26%
Fellowship training in sickle cell disease	19%		25%	11%
General fellowship training in hematology-oncology	35%		38%	47%
Attend sickle cell disease conferences regularly	52%		100%	N/A
Training in pain management	62%		81%	N/A
Training on sickle cell emergencies	N/A		100%	N/A
Primary care-based sickle cell disease experience (i.e., seen more than 10 patients in the primary care setting with sickle cell disease)	50%		75%	74%
Training on hydroxyurea management	85%		N/A	N/A
Training on transfusion decisions	90%		N/A	N/A
Training on screening for organ damage in sickle cell	80%		N/A	N/A
Awareness of psychosocial and cognitive issues in sickle cell	86%		N/A	N/A
*Question*: *How often should a sickle cell specialist be required to obtain Continuing Medical Education credit?*				
	**Round 1**	**Round 2**		**Round 3**
At least annually	33%	44%		16%
At least every 2 years	48%	56%		84%
Not Necessary	14%	0%		N/A
Other	5%	N/A		N/A

^1^ In Round 1 was asked in 2 questions: What type of knowledge/training must a provider have to be considered a SCD specialist? And What type of experience must a provider have to be considered a SCD specialist?

One area of discrepancy and follow-up discussion among the SCDIC members was whether an Advanced Primary Care provider (APC) could be considered a specialist. Based on the results of the first two rounds (including open-ended questions), we added a question in round 3 that asked, “If a patient sees an APC, is the patient considered affiliated with SCD care?” This question did not reach consensus, with only 47% of respondents answering affirmatively. Because we did not reach consensus, APCs are not included as specialists in our definition. Proponents of including APCs as specialists argued the benefits of having a specially SCD-trained APCs in PCP offices; these APCs could act as affiliates to a SCD network of providers (in contrast to a PCP without the specialized training).

## Discussion

The purpose of the current study was to obtain consensus using a Delphi process on the definition of “unaffiliation” from SCD care. Consensus was achieved for the following definitions of “unaffiliation from SCD care” and “sickle cell specialist”:

“An individual with SCD considered unaffiliated is someone who has not been seen by a sickle cell specialist in at least a year.

*A sickle cell specialist is someone with knowledge and experience in SCD*. *Having “knowledge” means*: *being knowledgeable of the 2014 NIH Guidelines*, *“Evidence-Based Management of SCD”*, *trained in hydroxyurea management and transfusions*, *trained on screening for organ damage in SCD*, *trained in pain management and on SCD emergencies*, *and is aware of psychosocial and cognitive issues in SCD*. *Experiences that are expected of a SCD specialist include experience working with SCD patients*, *mentored by a SCD specialist*, *regular attendance at SCD conferences*, *and obtains continuing medical education on SCD every 2 years*.*”* The type of conference or continuing medical education was not specified in this survey.

Obtaining consensus on these foundational terms is an important first step to advance the research and practice of quality care in SCD. Unaffiliated patients are likely a heterogeneous group of individuals that will require different strategies for affiliation and retention. Having a shared understanding of what it means to be unaffiliated will allow researchers to conduct studies that can optimize connecting diverse individuals with SCD to specialized care. This definition will allow researchers to quantify study endpoints and will also be used to support policies related to SCD care and the allocation of resources to connect patients to high-quality care.

Despite the contribution of this study to research and policy, we share a few cautions when applying this study to the realm of practice without further investigation. First, we recognize that the definition of SCD specialist has several areas that could be improved and clarified. One update is that the NIH 2014 guidelines were the only national guidelines in existence at the time this study was conducted. During this study, additional evidence-based guidelines emerged for the treatment of SCD sponsored by the ASH guidelines in 2019–2020. Further, the type of conference attendance required (including SCD-specific meetings) and the optimal continuing medical education necessary is needed.

Second, the purpose of this study was to advance SCD research, and we caution against strict adherence to the definitions in this study in clinical settings, particularly where “specialists” do not exist. We do not encourage current nonspecialist providers to remove individuals with SCD from their practices, unless successfully transferring to a specialist care provider. An alternative would be to acknowledge limitations of knowledge and either link to a SCD specialist or draw upon SCD treatment materials available through ASH. Existing models for linkages include: 1)“hub-and-spoke” models whereby SCD expertise is extended to areas without existing SCD specialists through the collaboration of nonspecialist PCPs or advance practice providers; 2) telemedicine in collaboration with local providers; or 3) intermittent care provided by specialists who travel to rural areas to support the care provided by primary care physicians between specialty visits [28,29]. In these models, patients may not be able to see specialists face to face but may be able to affiliate with them to help enhance care.

Similarly, nonspecialist providers might communicate indirectly with SCD specialists for affected patients, effectively imparting specialist-recommended care. Existing models include: (1) Community nonspecialist hematologists/oncologists with substantial numbers of benign hematology patients maintaining regular contact with academic SCD specialists on a regular basis (even if the patients are not seen at the academic medical center SCD clinic), along with attending annual ASH meetings, and using ASH resources; (2) Care provided by nonspecialists participating in successful SCD ECHO programs;(3) Routine care by nonspecialists with have their care guided by an SCD specialist at a local outreach clinic (e.g., Augusta University program); 4) Mentoring models whereby SCD specialists use telehealth or telemedicine to provide support to nonspecialists and direct patient-provider assistance; or 5) Co-management between pediatric primary health providers, pediatric specialty care, and future adult providers to support transitions for pediatric subpopulations [30]. This is an area of future work to better understand the effectiveness of various options for network providers.

Another practical consideration from this study is the question of how patients will know if their provider is an SCD specialist. We anticipate that patients are often unaware of the types of training and continuing education that their provider has, nor do they know their experiences working with SCD or knowledge of different SCD care strategies. It is our experience that patients are aware of educational background from brief overviews on websites or degrees handing on walls, but do not inquire deeply about the training experiences of the provider. This creates a loophole where we classify providers into specialist and non-specialist categories; but, are unable to connect patients to these providers. One potential solution is the establishment of a network, credential, or repository of sickle cell centers and specialist providers that can be used by patients to identify providers who can best meet their needs. This type of system should be developed through collaboration with SCD researchers, providers, implementation scientists, and the patient perspective to ensure a systematic approach where all potential risks and implications are considered. An example of potential risk is the situation of third-party payors limiting options for patients with SCD (based on these results); this may restrict all care for certain individuals who only have access to nonspecialists in their geographical region. Practical changes deriving from the definition should be accompanied by system-level changes that support access to quality care and avoid any new barriers to accessibility.

The impact of this work is to help lay a foundation of research that helps unaffiliated patients connect to care. We recognize however that *affiliation* is different from *engagement*. Engagement may be a more relevant construct for certain applications, particularly those that are patient-facing. A patient, for example, may consider themselves affiliated with care because they identify with a clinic, and know whom to call for an emergency or for particular needs (e.g., if they need to see a sickle cell specialist for preparation for surgery). Yet, they may not have attended clinic in a while based on perceived need. In this case, they may be affiliated but not engaged with care. Differentiating the overlapping constructs of affiliation and engagement, and how they translate to clinical outcomes separately and jointly, is an important area for future work.

While this study reached consensus among professionals with expertise in SCD, we recognize a limitation is that patient perspectives were not included. Patients with SCD are also often experts in their own disease with lived experiences. Obtaining alignment between the patient perspective and professional perspective will be important to build cohesion and improve care. As a follow-up to this study, the research team will be conducting a nationwide quantitative survey where we probe specific questions related to affiliation, agreement with professional opinion, and patients’ own self-identification of being affiliated (and engaged) or not. A particular area for further exploration will be on the duration of time since last appointment in relation to unaffiliation.

Another limitation of this study arises from the measurement of the criteria for SCD *specialists*. In this survey, we asked participants, “What type of knowledge/training and experience must a provider have to be considered a SCD specialist?” and provided a set of criteria with yes/no response options. The benefit of this approach was that it was easy for participants to complete in a rapid way and it helped us isolate which criteria were deemed most important among a pool of potential criteria. The limitation is that we are unable to determine whether *all* the criteria need to be met, or *some* of the criteria are sufficient (and which ones are necessary). To address this limitation, we facilitated multiple discussions with experts (including a focus group among Delphi participants) and feel confident that *all* criteria must be met to be considered a specialist. It was noted that most responders did check all of the boxes to indicate “all criteria” must be met but the question was not posed in this way. Simultaneously, we acknowledge that there may be variability in the types and quality of knowledge and experience, and this definition should not be interpreted as a hard rule. For example, if a provider meets all criteria except mentorship, then he or she may potentially still be considered a specialist provider. Similarly, although APCs were not deemed experts in this study, we hypothesize that an APC who meets all other criteria or who works closely with an MD SCD specialist in a network of care may be considered a specialist in another follow-up study. An additional limitation of our measurement was that we did not include “hematologist” as a potential category for “specialist”. This was by design because we were investigating providers that could be specialists beyond trained hematologists. We recognize that not all hematologists are specialists in SCD, however, and this is an area worthy of future inquiry. Distilling core versus more peripheral criteria is an important area of future work, particularly if a credential or provider network is developed.

## Conclusion

Although SCD is a rare disease, comprehensive care by providers who understand the uniqueness of the disease is needed. This poses a challenge in implementation because many providers simply do not have the requisite expertise to provide adequate care, leaving many patients at risk for negative health outcomes. This study advanced the field by establishing foundational terms that will support advanced research and practice in the care of SCD.
